# Estimating Trends in the Proportion of Transmitted and Acquired HIV Drug Resistance in a Long Term Observational Cohort in Germany

**DOI:** 10.1371/journal.pone.0104474

**Published:** 2014-08-22

**Authors:** Daniel Schmidt, Christian Kollan, Gerd Fätkenheuer, Eugen Schülter, Hans-Jürgen Stellbrink, Christian Noah, Björn-Erik Ole Jensen, Matthias Stoll, Johannes R. Bogner, Josef Eberle, Karolin Meixenberger, Claudia Kücherer, Osamah Hamouda, Barbara Bartmeyer, on behalf of the ClinSurv-HIV Drug Resistance Study Group in CHAIN

**Affiliations:** 1 Department of Infectious Disease Epidemiology, HIV/AIDS, STI and Blood Born Infections, Robert Koch-Institute, Berlin, Germany; 2 Clinic of Internal Medicine, University Köln, Köln, Germany; 3 ICH Study Centre, Hamburg, Germany; 4 Laboratory Lademannbogen, Hamburg, Germany; 5 Department of Gastroenterology, Hepatology and Infectious Diseases, Heinrich Heine University, Düsseldorf, Germany; 6 Clinic for Immunology and Rheumatology, Infectious Diseases Unit, Medical University Hannover, Hannover, Germany; 7 Department of Infectious Disease, Med IV, University Hospital of Munich, Munich Germany; 8 Max von Pettenkofer Institute, Institute of Virology, Ludwig Maximilians University, Munich, Germany; 9 Department of Infectious Diseases, HIV and Other Retroviruses, Robert Koch Institute, Berlin, Germany; University of British Columbia, Canada

## Abstract

**Objective:**

We assessed trends in the proportion of transmitted (TDR) and acquired (ADR) HIV drug resistance and associated mutations between 2001 and 2011 in the German ClinSurv-HIV Drug Resistance Study.

**Method:**

The German ClinSurv-HIV Drug Resistance Study is a subset of the German ClinSurv-HIV Cohort. For the ClinSurv-HIV Drug Resistance Study all available sequences isolated from patients in five study centres of the long term observational ClinSurv-HIV Cohort were included. TDR was estimated using the first viral sequence of antiretroviral treatment (ART) naïve patients. One HIV sequence/patient/year of ART experienced patients was considered to estimate the proportion of ADR. Trends in the proportion of HIV drug resistance were calculated by logistic regression.

**Results:**

9,528 patients were included into the analysis. HIV-sequences of antiretroviral naïve and treatment experienced patients were available from 34% (3,267/9,528) of patients. The proportion of TDR over time was stable at 10.4% (95% CI 9.1–11.8; p *_for trend_* = 0.6; 2001–2011). The proportion of ADR among all treated patients was 16%, whereas it was high among those with available HIV genotypic resistance test (64%; 1,310/2,049 sequences; 95% CI 62–66) but declined significantly over time (OR 0.8; 95% CI 0.77–0.83; p *_for trend_*<0.001; 2001–2011). Viral load monitoring subsequent to resistance testing was performed in the majority of treated patients (96%) and most of them (67%) were treated successfully.

**Conclusions:**

The proportion of TDR was stable in this study population. ADR declined significantly over time. This decline might have been influenced by broader resistance testing, resistance test guided therapy and the availability of more therapeutic options and not by a decline in the proportion of TDR within the study population.

## Introduction

Since the introduction of combination antiretroviral therapy the morbidity and mortality among people infected with HIV has been reduced dramatically [1]. However, antiretroviral treatment (ART) of HIV is still life-long, and the prolonged duration of therapy with emerging HIV drug resistance might leave many patients without treatment options. In recent years treatment options improved due to the approval of second generation drugs and new antiretroviral drug classes [2]. Moreover, fixed dose combinations were approved, increasing the level of adherence among patients [3], which also affects resistance development [4]. HIV drug resistance may be transmitted among recently infected patients within transmission chains but may also be acquired during non-suppressive antiretroviral treatment from ART experienced patients [5], [6]. In Germany, according to current treatment guidelines all patients should be genotyped routinely prior to ART initiation [7].

HIV drug resistance surveillance has been performed in many Western European countries within different study populations and settings. Most studies were performed in patients during primary HIV infection or in patients chronically infected with HIV prior to ART initiation [8]–[12]. Currently no epidemiological resistance data base exists, and no central repository for viral sequences is established in Germany. Studies on population level including all HIV sequences produced in routine clinical settings for estimating the proportion of TDR or ADR are not available. Therefore the aim of this study was to estimate trends in the proportion of overall TDR and ADR and within different drug classes between 2001 and 2011, as well as single mutations within the German ClinSurv-HIV Drug Resistance Study. This is the first time that viral sequence data is linked with epidemiological and antiretroviral treatment data in a large resistance study in Germany including ART naïve as well as treated patients in order to estimate the proportion of transmitted and acquired HIV drug resistance.

## Materials and Methods

### The ClinSurv-HIV Cohort, study design

The ClinSurv-HIV project protocol was approved by the German Federal Commissioner for Data Protection. ClinSurv-HIV is an ongoing, prospective, long-term observational cohort study. The study design has been described in detail elsewhere [13]. In brief, 15 clinical centres in different, predominantly urban areas in Germany are involved in the study and consecutively monitored subjects since January 1^st^ 1999. The cohort comprises all individuals infected with HIV in the participating clinical centres. Only basic data are reported every six months. The data set is anonymized and comprises demographic data, time-related variables on clinical events, AIDS-defining diagnoses, and detailed data on antiretroviral treatment. Patients with available sequences were identified at the five ClinSurv-HIV Drug Resistance Study centres participating in this analysis. Any identifying information was removed at the local study centres, the sequences were labelled with a new identifier allowing linkage with anonymized patient data in the ClinSurv data base. All data analyses were performed on the anonymized data set.

The Robert Koch Institute (RKI) is the German national public health institute, therefore the Federal Commissioner for Data Protection is the responsible entity for studies which are conducted by the Robert Koch Institute. Information on HIV infection collected in ClinSurv corresponds to the data reported to the RKI according to legal requirements implemented by the national Protection against Infection act (IfSG) of 2001. All patient data collected in ClinSurv are generated during routine care. The German Federal Commissioner for Data Protection therefore waived the need for ethical approval for the ClinSurv study. No written informed consent is required from patients.

### ClinSurv-HIV Drug Resistance Study

For the ClinSurv-HIV Drug Resistance substudy, all patients infected with HIV under care in five study sites (University of Cologne, University of Düsseldorf, University of Hanover, University of Munich, ICH Study Centre Hamburg) of the ClinSurv-HIV Cohort study group with at least one HIV genotypic resistance analysis result were identified. The nucleotide sequences were processed through the Stanford University Genotypic Resistance Interpretation Algorithm (www.hivdb.stanford.edu; HIVdb version 6.2.0; 2012) in order to identify amino acid substitutions and to determine drug susceptibility. For epidemiological analysis, HIV sequences isolated from ART naïve patients were analysed using the surveillance HIV drug resistance mutation list, SDRM [14]. Only the first HIV genotypic resistance test per patient while treatment naïve was considered for the estimation of the prevalence of TDR. HIV sequences isolated from ART experienced patients were analysed using the mutation list of the International Antiviral Society-USA, IAS, 2011 [15]. Overall ADR was estimated by including one HIV sequence/patient/year from antiretroviral treatment experienced patients. For the estimation of ADR within different drug classes only viral sequences of patients treated with the respective drug class were considered. HIV subtype on patient level was assigned based on the first available viral sequence of a patient centrally using the REGA HIV-1 Subtyping Tool - Version 2.0 [16].

### Statistical analysis

Viral sequences available between 1998 and 2011 were collected and analysed. For the estimation of trends in the proportion of HIV drug resistance over time viral sequences sampled between 2001 and 2011 were included into the analyses. The characteristics of patients with available sequences compared to those who were not genotyped were compared with simple logistic regression. Patients and viral sequences were categorised into ART naïve and treatment experienced. Viral sequences were considered to originate from treatment naïve patients in case of ART start ≤15 days prior to the date of resistance testing, to account for delays in the documentation of the date of resistance testing results and the actual date of blood sampling. The time between resistance testing and antiretroviral treatment start and the proportion of patients with resistance test before ART start were analysed by using data from patients with documented first-line treatment start. The proportion of patients with antiretroviral treatment failure undergoing resistance testing was calculated for patients with first line treatment start in one of the resistance study centres who had >180 days of treatment experience and a resistance test within 90 days after virologic failure. Virologic failure was defined as two consecutive viral load measurements with >50 copies/ml within 180 days or one viral load measurement with >1000 copies/ml. For different drug classes the proportion of cumulative ART exposure per year, the median days of previous ART exposure and the proportion of patients showing history of exposure to the respective drug class were calculated, excluding treatment interruption time. A simple linear regression was performed on the duration of antiretroviral treatment exposure in days prior to resistance testing with increasing year of genotyping for different drug classes and on the total treatment exposure. In a univariate analysis of factors associated with HIV drug resistance the following covariates were separately included: age, gender, transmission group category, HIV subtype, ART interruption, duration of previous ART exposure, CD4 cell count, and plasma viral load at the time point of testing or 30 days prior to or 15 days after testing, year of resistance test at documented ART start. Factors significantly associated with acquired HIV drug resistance in a univariate analysis were included into multiple logistic regression. For the analysis of factors associated with acquired HIV drug resistance only the last sequence of ART experienced patients was included. The proportion of patients showing a history of exposure to a drug class, trends in the proportion of patients with ART interruption, gender, transmission group category, HIV subtype were calculated by simple logistic regression. Median plasma viral loads and CD4 cell counts were calculated for ART naïve and treated patients. The Mann-Whitney-U-test (MWT) was used to compare viral loads, CD4 cell counts and the age at ART start for patients with resistant and susceptible HIV. Confidence intervals were calculated using a Wilson score confidence interval. Trends in the prevalence of HIV drug resistance mutations, DRMs, (*p_for trend_*) were calculated by logistic regression. All p-values were two sided, and a p-value of <0.05 was considered significant. Data were analysed using SPSS 18.0.3 and R 2.12.1.

## Results

A total of 9,528 ClinSurv patients were enrolled in the five study centers participating in the resistance study. 4,989 viral sequences were collected from 34% (3,267/9,528) of these patients. Nearly half of these HIV sequences (47%; 2,365/4,989) were generated while patients were ART naïve, the other half of viral sequences (50%; 2,495/4,989) were produced while patients were treated with antiretroviral drugs. The ART status of the patients could not be clarified for 3% (129/4,989) of the HIV sequence data. Of the patients who have not been genotyped (n = 6,261), 4,895 patients were seen in the resistance study centres while ART naïve, and of 5,262 patients data were collected while treated with antiretroviral drugs (Table 1). In total 86% (8,165/9,528) of the patients in the five Resistance Study centres were ART experienced. Among all patients who have been treated, HIV drug resistance was identified in 16% (1,347/8,165) of patients.

**Table 1:** Characteristics of patients in the ClinSurv- HIV Drug Resistance Study centers and of patients with available resistance test.

	Resistance Study centres, patients without resistance test						Patients with resistance test								
Patients	Total number of patients		ART naïve group		ART experienced		Total number of patients		Total number of sequences		Sequences from naïve patients		Sequences from treated patients		p value
Total, n (%)	6261	(100,0%)	4895	(100,0%)	5262	(100,0%)	3267	(100,0%)	4989	(100,0%)	2365	(100,0%)	2495	(100,0%)	
**Median age at enrolment in years (IQR)**	36,5	(30,5–43,9)	36,0	(29,9–43,2)	37,0	(31,1–44,5)	36,8	(30,3–44,1)	36,7	(30,3–44,0)	37,0	(30,1–44,1)	36,4	(30,3–44,0)	0.765
**Median age at genotyping in years (IQR)**							39,6	(33,0–47,0)	40,2	(33,6–47,6)	38,0	(31,0–45,0)	42,4	(36,6–49,8)	
**Gender, n (%)**															
Men	5110	(81,6%)	3969	(81,1%)	4.310	(81,9%)	2683	(82,1%)	4028	(80,7%)	1966	(83,1%)	1967	(78,8%)	
Women	1151	(18,4%)	926	(18,9%)	952	(18,1%)	584	(17,9%)	961	(19,3%)	399	(16,9%)	528	(21,2%)	0.518
**Mode of HIV transmission, n (%)**															
Men who have sex with men	3575	(57,1%)	2770	(56,6%)	3.030	(57,6%)	1904	(58,3%)	2776	(55,6%)	1405	(59,4%)	1316	(52,7%)	
Heterosexuals	729	(11,6%)	610	(12,5%)	617	(11,7%)	445	(13,6%)	673	(13,5%)	346	(14,6%)	311	(12,5%)	0.041
High prevalence country	656	(10,5%)	513	(10,5%)	565	(10,7%)	405	(12,4%)	712	(14,3%)	276	(11,7%)	418	(16,8%)	0.034
Intravenous drug use	551	(8,8%)	434	(8,9%)	435	(8,3%)	180	(5,5%)	249	(5,0%)	77	(3,3%)	162	(6,5%)	**<0.001**
Others	89	(1,4%)	59	(1,2%)	77	(1,5%)	38	(1,2%)	93	(1,9%)	24	(1,0%)	53	(2,1%)	0.209
Unknown	661	(10,6%)	509	(10,4%)	538	(10,2%)	295	(9,0%)	486	(9,7%)	237	(10,0%)	235	(9,4%)	0.019
**Region of origin, n (%)**															
Germany	4729	(75,5%)	3706	(75,7%)	3.990	(75,8%)	2416	(74,0%)	3574	(71,6%)	1783	(75,4%)	1699	(68,1%)	
Africa, Near East	571	(9,1%)	439	(9,0%)	486	(9,2%)	379	(11,6%)	679	(13,6%)	252	(10,7%)	410	(16,4%)	**<0.001**
Western Europe	242	(3,9%)	182	(3,7%)	199	(3,8%)	113	(3,5%)	177	(3,5%)	70	(3,0%)	103	(4,1%)	0.441
Central Europe	201	(3,2%)	164	(3,4%)	171	(3,2%)	123	(3,8%)	192	(3,8%)	92	(3,9%)	100	(4,0%)	0.124
Asia	152	(2,4%)	123	(2,5%)	136	(2,6%)	74	(2,3%)	129	(2,6%)	61	(2,6%)	64	(2,6%)	0.738
America, Oceania	145	(2,3%)	106	(2,2%)	122	(2,3%)	65	(2,0%)	89	(1,8%)	44	(1,9%)	44	(1,8%)	0.388
Eastern Europe	85	(1,4%)	70	(1,4%)	62	(1,2%)	54	(1,7%)	72	(1,4%)	48	(2,0%)	21	(0,8%)	0.215
Unknown	136	(2,2%)	105	(2,1%)	96	(1,8%)	43	(1,3%)	77	(1,5%)	15	(0,6%)	54	(2,2%)	0.007
**Calender year of genotyping, n (%)**															
1998–2000							53	(1,6%)	58	(1,2%)	4	(0,2%)	42	(1,7%)	
2001–2004							632	(19,3%)	920	(18,4%)	265	(11,2%)	603	(24,2%)	
2005–2008							1523	(46,6%)	2265	(45,4%)	1039	(43,9%)	1181	(47,3%)	
2009–2011							1059	(32,4%)	1746	(35,0%)	1057	(44,7%)	669	(26,8%)	
**HIV-1 Subtype, n (%)**							**first sequence**		**for each sequence**						
Subtype B							2240	(68,6%)	3397	(68,1%)	1574	(66,6%)	1733	(69,5%)	
Subtype A							281	(8,6%)	432	(8,7%)	220	(9,3%)	198	(7,9%)	
Subtype C							98	(3,0%)	157	(3,1%)	77	(3,3%)	76	(3,0%)	
HIV-1 CRF 02_AG							96	(2,9%)	148	(3,0%)	75	(3,2%)	71	(2,8%)	
Subtype G							53	(1,6%)	105	(2,1%)	47	(2,0%)	54	(2,2%)	
Subtype D							27	(0,8%)	42	(0,8%)	23	(1,0%)	19	(0,8%)	
HIV-1 CRF 06_CPX							24	(0,7%)	40	(0,8%)	16	(0,7%)	23	(0,9%)	
Subtype F							12	(0,4%)	15	(0,3%)	8	(0,3%)	5	(0,2%)	
Other HIV-1 subtypes							13	(0,4%)	29	(0,6%)	10	(0,4%)	19	(0,8%)	
Not typeable							423	(12,9%)	624	(12,5%)	315	(13,3%)	297	(11,9%)	
**CD4 count (cells/µl) at presentation, n (%)**															
<50	526	(8,4%)					316	(9,7%)							
50–200	1115	(17,8%)					649	(19,9%)							
>200–350	1137	(18,2%)					737	(22,6%)							
>350–500	1080	(17,2%)					586	(17,9%)							
>500	1527	(24,4%)					700	(21,4%)							
Missing	876	(14,0%)					279	(8,5%)							
**Median CD4 count (IQR; range)**	340	(161–536; 0–3231)					310	(147–490; 0–2946)							

IQR: interquartile ranges;

Other HIV-1 subtypes included subtype J (n = 1) and the circulating recombinant forms: 05_DF, 01_AE, 11_CPX, 03_AB, 13_CPX, 10_CD, 12_BF, 18_cpx, 27_cpx, 43_02G;

### Characteristics of patients with available sequences

Patients were predominantly male (82%; 2,683/3,267); 18% (584/3,267) were female. Median age at the time point of HIV genotypic resistance testing was 40 years (33.0–47.0). Median CD4 cell count at first visit was 310 cells/µl (IQR: 147–490). The main transmission group category was sex between men (58%; 1,904/3,267), followed by heterosexual contacts (14%; 445/3,267) and by patients originating from high-prevalence countries (12%; 405/3,267). Median time between first HIV genotypic resistance test and ART start was 33 days (IQR: 13–169). The proportion of patients with resistance test before ART start in the five study centres increased from 0.4% in 2000 to 69% in 2009 and declined thereafter to 46% in 2010 and 21% in 2011. The duration between HIV diagnosis and first resistance test was in median 45 days (IQR: 16–481) for ART naïve patients.

The characteristics of patients with resistance test and those without differed as follows: within the category risk of transmission in the Resistance study group we observed more patients with heterosexual contacts (with resistance test: 13.6% vs. 11.6% without test, p = 0.04), more patients from high prevalence countries (with resistance test: 12.4% vs. 10.5% without test, p = 0.03) and fewer patients with intravenous drug use (with resistance test: 5.5% vs. 8.8% without test, p<0.001); within the category region of origin in the Resistance study group we observed more patients being from Africa, Near East (with resistance test: 11.6% vs. 9.1% without test) (Table 1). Patients were predominantly infected with HIV-1 subtype B strains (69%; 2,240/3,267), but 18% (604/3,267) of patients harbored a non-B subtype infection. For 13% (423/3,267) of patients the HIV-1 subtype could not be determined by REGA HIV-1 Subtyping Tool. HIV-1 subtype A was most prevalent among the non-B subtypes (9%), followed by circulating recombinant forms (4%) (Table 1). The majority of women were infected with HIV-1 non-B subtypes (non-B subtypes: 53%; 312/584 *vs.* subtype-B: 33%; 192/584), predominantly subtype A (24%; 140/584). Nearly half of the female study population originated from high-prevalence countries: 46% (270/584). At time point of HIV genotypic resistance testing, the median viral load (VL) for ART naïve patients was 4.73 log_10_ copies/ml (IQR: 4.1–5.3), median CD4 cell count was 285 cells/µl (IQR: 151–437). Both VL and CD4 cell counts did not differ significantly between patients infected with susceptible or resistant viruses (VL susceptible: 4.74, IQR: 4.1–5.3 *vs.* VL resistant: 4.69, IQR: 4.2–5.3, p = 0.93; CD4 susceptible: 286, IQR: 150–437 vs. CD4 resistant: 274, IQR: 169–443, p = 0.90). The median VL of treated patients with detectable plasma virus was 4.02 log_10_ copies/ml (IQR: 3.1–4.8), median CD4 cell count of treated patients was 271 cells/µl (IQR: 150–423). VL and CD4 cell count did not differ significantly between patients infected with susceptible or resistant viruses (VL susceptible: 4.13, IQR: 2.8–4.9 *vs.* VL resistant: 3.96, IQR: 3.2–4.7, p = 0.50; CD4 susceptible: 280, IQR: 160–430 vs. CD4 resistant: 266, IQR: 145–420, p = 0.29).

### Transmitted HIV drug resistance, TDR

**Figure 1:**
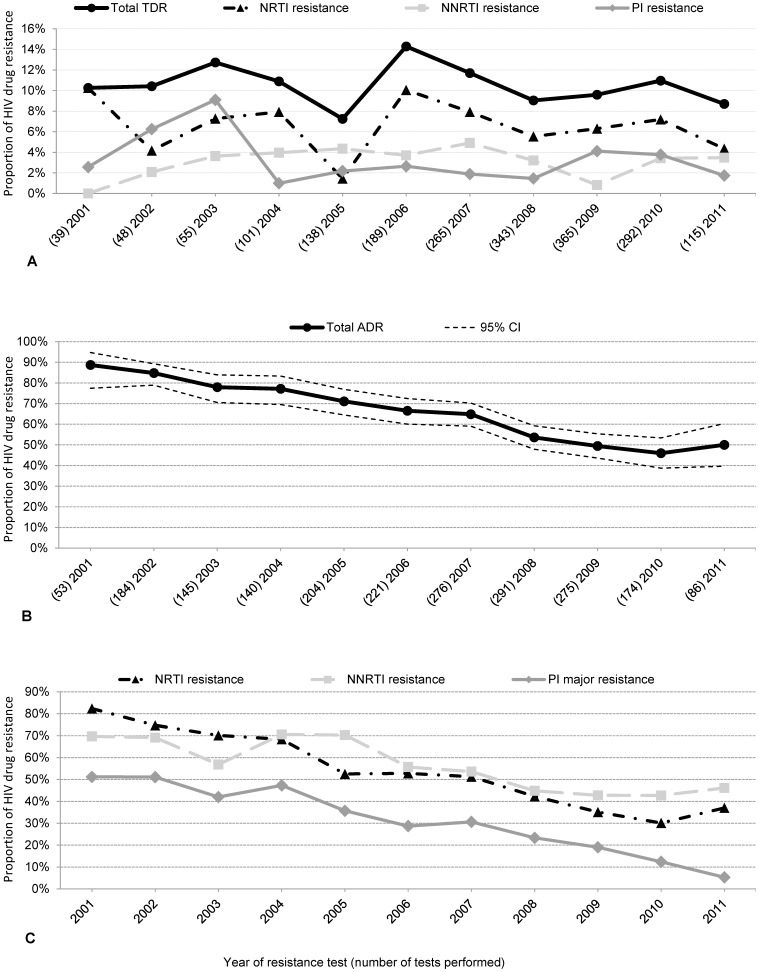
Proportion of HIV drug resistance in sequences from treatment naïve patients and treatment experienced patients between 2001 and 2011. **A** Proportion of HIV drug resistance was determined using the first Prot/RT sequences from treatment naïve patients (n = 1950) by year according to the SDRM mutation list (Bennett et al. 2009). The proportion of TDR over time was stable at 10.4% (95% CI 9.1–11.8; p *_for trend_* = 0.6; 2001–2011). **B** Proportion of overall ADR (64%; 1,310/2,049 sequences; 95% CI 62–66) declined significantly over time (OR 0.8; 95% CI 0.77–0.83; p for trend<0.001; 2001–2011) in sequences from treated patients (n = 2,049) according to IAS mutation list 2011. **C** Proportion of ADR within different antiretroviral drug classes declined for all classes (NNRTI 55%, NRTI 51%, PI 30%; p for trend<0.001; 2001–2011) in sequences from patients treated with the respective drug class according to IAS mutation list 2011.

Overall TDR according to SDRM list was identified among the first HIV sequences available before ART initiation in 10.4% (203/1,950; 95% CI 9.1–11.8) and remained stable over time (OR: 0.98; p *_for trend_* = 0.6; 2001–2011) (Figure 1A). Nucleoside reverse transcriptase inhibitor (NRTI) resistance was detected in 7% (128/1,950; 95% CI 6–8), followed by 3% (61/1,950; 95% CI 2–4) non-nucleoside reverse transcriptase inhibitor (NNRTI) resistance and 3% (56/1,950; 95% CI 2–4) protease inhibitor (PI) resistance. The prevalence of thymidine analogue mutations (TAMs) was 5% (89/1,950; 95% CI 4–6), and revertant mutations at position 215 of the reverse transcriptase (RT) were found in 3% (56/1,950; 95% CI 2–4) of first viral strains analysed from ART naïve patients (Table 2).

**Table 2:** Prevalence of transmitted HIV drug resistance according to the SDRM mutation list and of acquired HIV drug resistance according to the IAS mutation list.

Transmitted HIV drug resistance	n (%)	(95% CI)	Beta	OR (95% CI)	p *for trend*
First Prot/RT sequence from naive patients	Prevalence of DRMs according to SDRM mutation list (Bennett D. et al. 2009)				
Total	1950 (100.0%)				p *(2001–2011)*
DRMs	203 (10.4%)	(9.1–11.8)	−0.018	0.98 (0.92–1.04)	0.561
NRTI mutations	128 (6.6%)	(5.5–7.8)	−0.016	0.98 (0.91–1.06)	0.667
TA mutations	89 (4.6%)	(3.7–5.6)	0.002	1.00 (0.92–1.10)	0.972
T215revertants	56 (2.9%)	(2.2–3.7)	0.058	1.06 (0.94–1.19)	0.337
NNRTI resistance	61 (3.1%)	(2.4–4.0)	−0.031	0.97 (0.87–1.08)	0.560
PI resistance	56 (2.9%)	(2.2–3.7)	−0.020	0.98 (0.88–1.10)	0.723
Single/multi drug class resistance	203 (10.4%)				
One class resistance	169 (8.7%)	(7.5–10.0)			
Two classes resistance	26 (1.3%)	(0.9–1.9)			
Three classes resistance	8 (0.4%)	(0.2–0.8)			

Acquired HIV drug resistance	n (%)	(95% CI)	Beta	OR (95% CI)	p *for trend*
Prot/RT sequences from treated patients	Prevalence of DRMs according to IAS list (Johnson V. et al. 2011)				
Total	2049 (100.0%)				p *(2001–2011)*
DRMs	1310 (63.9%)	(61.8–66.0)	−0.220	0.80 (0.77–0.83)	**<0.001**
Subgroup of NRTI treated patients	1958 (100.0%)				
NRTI mutations	1007 (51.4%)	(49.2–53.6)	−0.224	0.80 (0.77–0.83)	**<0.001**
TA mutations	696 (35.5%)	(33.5–37.7)	−0.216	0.81 (0.78–0.84)	**<0.001**
T215revertants	72 (3.7%)	(2.9–4.6)	0.001	1.00 (0.92–1.09)	0.990
Subgroup of NNRTI treated patients	1333 (100.0%)				
NNRTI resistance	730 (54.8%)	(52.1–57.4)	−0.144	0.87 (0.83–0.90)	**<0.001**
Subgroup of PI treated patients	1586 (100.0%)				
PI resistance	473 (29.8%)	(27.6–32.1)	−0.227	0.80 (0.76–0.83)	**<0.001**
Single/multi drug class resistance	1310 (63.9%)				
One class resistance	490 (23.9%)	(22.1–25.8)			
Two classes resistance	500 (24.4%)	(22.6–26.3)			
Three classes resistance	320 (15.6%)	(14.1–17.3)			
**Integrase sequences from treated patients**	**150 (100.0%)**				**p ** ***(2006–2011)***
INI resistance	10 (6.7%)	(3.7–11.8)			
INI resistance on INI treated (n = 33)	10 (30.3%)	(17.4–47.3)	−0.571	0.57 (0.30–1.06)	0.077

Highly significant results are marked in bold fonts.

### Acquired HIV drug resistance, ADR

**Figure 2:**
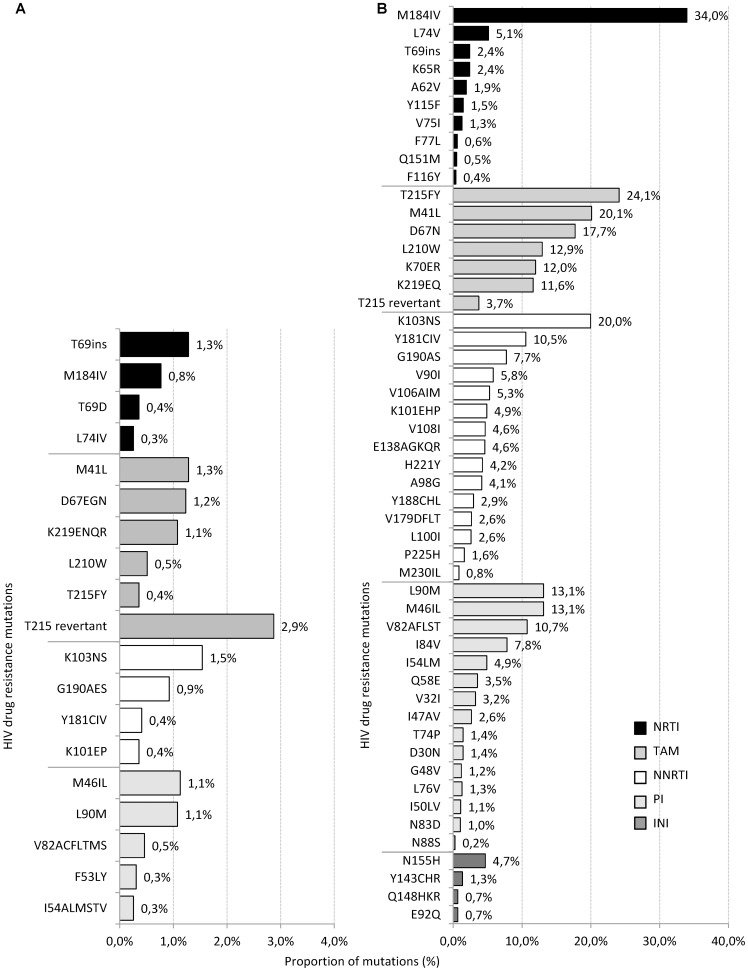
Proportion of resistance mutations in sequences from treatment naïve and treatment experienced patients identified between 2001 and 2011. **A** Proportion of resistance mutations in first Prot/RT sequence from treatment naïve patients (n = 1950; mutations with ≥0.3% shown) according to SDRM mutation list (Bennett 2009 et al.). **B** Proportion of resistance mutations in sequences from treated patients (Prot/RT n = 2,049; Int n = 150) according to IAS mutation list 2011. Bars in black: NRTI mutations, grey bars: TAMS, white bars: NNRTI mutations, bars in light grey: PI mutations, dark grey bars: INI mutations.

ADR was calculated using maximal one HIV sequence per year of antiretroviral treatment experienced patients. Overall ADR was high (64%; 1,310/2,049 sequences; 95% CI 62–66) but declined significantly over time (OR 0.8; 95% CI 0.77–0.83; p*_for trend_*<0.001; 2001–2011) (Figure 1B). To estimate HIV drug resistance in different drug classes, only viral sequences isolated from those patients who received the respective drug class were included into the analysis. Predominantly NNRTI resistance was identified (55%; 730/1333; 95% CI 52–57), followed by NRTI resistance in 51% (1,007/1,958; 95% CI 49–54) and PI resistance in 30% (473/1586; 95% CI 28–32). The proportion of ADR declined significantly over time for all three drug classes (p*_for trend_*<0.001; 2001–2011) (Figure 1C). INI resistance was detected in 30% (10/33; 95% CI 17–47) of INI treated patients corresponding to 7% (10/150; 95% CI 4–12) among all HIV integrase sequences of ART experienced patients (Table 2). The most prevalent NRTI associated mutation identified among ART experienced patients was M184IV (34%). The most common TAMs were T215FY (25%), M41L (21%) and D67N (18%). The prevalence of PI mutations I84V and I54LM associated with darunavir and atazanavir resistance were 8% and 5%, respectively. The PI associated resistance mutations M46L and L90M were most prevalent with 14% (Figure 2).

### Factors associated with acquired HIV drug resistance

Factors significantly associated with a lower risk of ADR by using a univariate model were being female compared to male, transmission group category IDU compared to MSM, being infected with non-B subtype compared to subtype B, reported ART interruption at genotyping compared to reported continuous antiretroviral treatment at the time of genotyping and the cumulative duration of ART interruption (Table 3). In a multiple logistic regression significant factors from the univariate analysis were included into the model. Factors significantly associated with a lower risk of ADR were being female compared to male, reported ART interruption compared to reported continuous antiretroviral treatment at the time of genotyping, increasing calendar year of genotyping and the cumulative duration of ART interruption (Table 4). The duration of antiretroviral treatment was associated with a higher risk of ADR in both the univariate and multiple logistic regression.

**Table 3:** Univariate analysis of factors associated with HIV drug resistance (IAS list 2011) among ART experienced patients (last sequence).

	Total	resistant HIV	susceptible HIV	OR (95% CI)	p-Value
**Treatment experienced patients, n (%)**	1437 (100%)	857 (59.6%)	580 (40.4%)		
**Median age at ART start, years (IQR)**	36.2 (30.1–43.4)	35.8 (30.4–43.3)	36.4 (29.7–43.6)		0.630^a^
**Sex, n (%)**					
Men	1143 (79.5%)	709 (82.7%)	434 (74.8%)	1	
Women	294 (20.5%)	148 (17.3%)	146 (25.2%)	0.62 (0.48–0.80)	**<0.001** b
**Mode of HIV transmission, n (%)**					
Men who have sex with men	784 (54.6%)	483 (56.4%)	301 (51.9%)	1	
High prevalence country	210 (14.6%)	117 (13.7%)	93 (16.0%)	0.78 (0.58–1.07)	0.121^b^
Heterosexuals	180 (12.5%)	114 (13.3%)	66 (11.4%)	1.08 (0.77–1.51)	0.667^b^
Intravenous drug use	111 (7.7%)	56 (6.5%)	55 (9.5%)	0.64 (0.43–0.95)	0.025^b^
Others	21 (1.5%)	14 (1.6%)	7 (1.2%)	1.25 (0.50–3.12)	0.638^b^
Unknown	131 (9.1%)	73 (8.5%)	58 (10.0%)	0.78 (0.54–1.14)	0.203^b^
**HIV subtype, n (%)**					
Subtype B	1007 (70.1%)	637 (74.3%)	370 (63.8%)	1	
Subtype non-B	263 (18.3%)	133 (15.5%)	130 (22.4%)	0.59 (0.45–0.78)	**<0.001** b
Not typeable	167 (11.6%)	87 (10.2%)	80 (13.8%)	0.63 (0.45–0.88)	0.006^b^
**Treatment status at genotyping, n (%)**					
Under treatment	1091 (75.9%)	726 (84.7%)	365 (62.9%)	1	
Pause	293 (20.4%)	102 (11.9%)	191 (32.9%)	0.27 (0.21–0.35)	**<0.001** b
Missing	53 (3.7%)	29 (3.4%)	24 (4.1%)	0.61 (0.35–1.06)	0.079^b^
**Treatment exposure time, per year**				1.13 (1.11–1.17)	**<0.001** b
**Duration of interruption, per year**				0.84 (0.79–0.89)	**<0.001** b
**Viral load at genotyping, n (%)**	1151 (80.1%)	688 (80.3%)	463 (79.8%)		
Median HIV-RNA log10 cps/ml (IQR)	3.94 (2.98–4.76)	3.87 (3.09–4.61)	4.11 (2.80–4.88)		0.181^a^
**CD4 cells at genotyping, n (%)**	1097 (76.3%)	659 (76.9%)	438 (75.5%)		
Median CD4 cells/µl (IQR)	280 (164–434)	278 (161–435)	286 (166–430)		0.721^a^

IQR: interquartile ranges;

CI: 95% confidence intervals;

a Mann-Whitney-U-Test;

b simple logistic regression;

Highly significant results are marked in bold fonts;

**Table 4:** Multiple regression analysis of factors associated with HIV drug resistance (IAS list 2011) among ART experienced patients (last sequence).

	Total	resistant HIV	susceptible HIV	OR (95% CI)	p-Value
**Treatment experienced patients, n (%)**	1437 (100%)	857 (59.6%)	580 (40.4%)		
**Sex, n (%)**					
Men	1143 (79.5%)	709 (82.7%)	434 (74.8%)	1	
Women	294 (20.5%)	148 (17.3%)	146 (25.2%)	0.71 (0.53–0.94)	0.017
**Treatment status at genotyping, n (%)**					
Under treatment	1091 (75.9%)	726 (84.7%)	365 (62.9%)	1	
Pause	293 (20.4%)	102 (11.9%)	191 (32.9%)	0.32 (0.24–0.42)	**<0.001**
Missing	53 (3.7%)	29 (3.4%)	24 (4.1%)	0.61 (0.34–1.10)	0.102
**Calendar year of resistance test (2001–2011)**				0.80 (0.76–0.84)	**<0.001**
**Treatment exposure time, per year**				1.13 (1.10–1.16)	**<0.001**

Treatment exposure time is the accumulated time documented for a patient to receive antiretroviral therapy, excluding times of interruption.

To investigate the reduced risk for women to carry resistant viral strains, the duration of previous ART exposure was analysed. Results showed, that women had a significantly lower ART exposure time than men (women median 3.9 years, IQR 0.8–7.1 to men median 5.0, IQR 1.3–9.1, respectively; OR 0.95, 95%CI 0.92–0.98, p<0.001).

### Treatment exposure

The majority of antiretroviral treated patients (98%; 1,956/1,987) showed a history of exposure to NRTIs, 67% (1,333/1,987) to NNRTIs, 80% (1,584/1,987) to PIs and 5% (103/1,987) to INIs.

Over time there was a small but significant decrease in the proportion of patients with exposure to NRTI (from 100% in 2001 to 98% in 2011; OR 0.78; 95% CI 0.67–0.91; p = 0.002), NNRTI use remained stable during the period of observation (from 65% in 2001, peaking in 2007 at 76% and leveling off thereafter with 63% in 2011; OR 1.0; 95% CI 0.96–1.0; p = 0.92). PI exposure increased significantly over time (from 80% in 2001 to 90% in 2011; OR 1.1; 95% CI 1.0–1.1; p = 0.009). INI use increased significantly over time (from 3% in 2007 to 25% in 2011; OR 2.0; 95% CI 1.7–2.3; p<0.001).

The simple linear regression showed a non-significant slight increase in the duration of ART exposure in days prior to resistance testing with increasing year of genotyping for NRTI (R^2^: 0.00; coefficient B: 6.6; 95% CI −19–32; p = 0.62), a significant increase for NNRTI (R^2^: 0.007; coefficient B: 26.6; 95% CI 13–40; p<0.001), for PI (R^2^: 0.006; coefficient B: 35.8; 95% CI 16–55; p<0.001) and for INI (R^2^: 0.045; coefficient B: 8.6; 95% CI 7–10; p<0.001). The duration of total ART exposure prior to resistance testing increased but not significantly over time (R^2^: 0.001; coefficient B: 22.8; 95% CI −3.3–49; p = 0.087).

Considering the regimen at resistance testing there was an increase in boosted PI containing regimens, especially for ritonavir boosted NRTI/PI combinations (from 10% in 2001 to 46% in 2011, NRTI+PI, overall 35%). PI regimens without booster almost disappeared over time. Triple class combinations like NRTI/NNRTI/PI declined over time whereas newer drug class containing regimens increased (from 0.6% in 2002 to 21% in 2011, overall 7%). Combination of NRTI/NNRTI showed a decline over time (from 29% in 2001 to 20% in 2010 and only 10% in 2011, overall 20%). Treatment interruption at the time of resistance testing was highly frequent in this study population but remained stable over time (17% in 2002 to 17% in 2011, overall 20%; OR 1.04; 95% CI 1.0–1.1; p *_for trend_* = 0.073; 2002–2011).

The proportion of patients undergoing resistance testing within 90 days after failing ART in the five study centres increased from 2.2% in 2001 to 21% in 2010 and amounted to 13% in 2011.

Treatment success post resistance testing was observed for 67% (928/1,386) of ART experienced patients. HIV drug resistance was observed in 60% (828/1,386) of this study population whereas 40% (558/1,386) showed no resistance associated mutations. However, the proportion of patients with successful viral suppression after resistance testing did not differ between those patients with susceptible and resistant HIV strains (67%; 553/828 resistant HIV vs. 67%; 375/558 susceptible HIV). A switch of therapy between different drug classes was observed significantly more frequent for patients harbouring resistant strains (64%; 530/828) than for patients with susceptible viruses (50%; 279/558).

## Discussion

Results of this study showed that the estimated prevalence of TDR over time among ART naïve patients remained stable at a high level, whereas overall prevalence of ADR as well as drug resistance within different drug classes in patients under antiretroviral treatment declined significantly over time. The decline of ADR could be influenced by several factors. Presumably antiretroviral drug related effects like enhanced treatment optimization and resistance test guided therapy as well as broader resistance testing in the study population are considered to be reasonable factors influencing this decline.

The high but stable level of TDR is comparable to other prevalence estimates in long term observational cohorts in Germany as well as in other European countries [8], [17], [18] and to cohorts with patients chronically infected with HIV in other Western European countries [17]–[19]. Since the date of HIV infection is not known in this study population, the estimates of TDR might be underestimates as in many cases reversion to wild type virus might have occurred. However, the proportion of TDR observed in antiretroviral treatment naïve patients (10.4%) was comparable to those observed in German HIV-1 seroconverters (12%) [8], [12]. The proportion of TDR in previously untreated patients reflects transmission of resistant strains at the time-point of infection mostly some years ago with the risk that primary transmitted mutations at the time of resistance testing are not visible anymore. This requires regular monitoring and evaluation of TDR. The most frequent mutations observed in ART naïve patients were T215 revertants which were transmitted as revertant or evolved from viruses harbouring a T215F or T215Y mutation. As also reported by studies from other countries [20] TAMs were the predominant single mutations determined in this study population despite changing prescription policies regarding stavudine and zidovudine, which select for these mutations. One possible explanation for this phenomenon might be that frequently used drugs like abacavir and tenofovir maintain the prevalence of such mutations in patients receiving antiretroviral therapy, and mutations may be transmitted in case of insufficient virus suppression [21], [22]. In contrast, the proportion of ADR in antiretroviral treatment experienced patients at the time point of resistance testing declined significantly over the period of observation in this study population, as also observed in other Western European study populations [23]. This decline was not related to a lower proportion of TDR in treatment naïve patients. The proportion of ADR among the treated population could be influenced by the time of drug exposure to different drug classes, the number of substances ever used, and the time of ART interruption. The risk of ADR was significantly lower in patients with documented ART interruption at the time of genotyping than in those patients with continuous treatment as showed by the univariate and multivariate analysis of factors associated with the risk of ADR. The reason might be that in the absence of selective drug pressure, secondary resistant mutations are rapidly overgrown by wild type virus [24]. Although studies of ART interruption have demonstrated the potential for negative clinical consequences like increased risk of death and serious AIDS defining events [25], non-structured ART interruptions are common in daily clinical routine and the proportion of patients who interrupted therapy remained stable over time at a high level (21%) as shown in this study.

As expected the time a patient was exposed to ART prior to resistance testing was significantly associated with a higher risk of ADR in the univariate and multivariate analysis. The significantly lower risk of ADR for women was most likely caused by their significantly shorter duration of ART exposure prior genotyping.

Regarding the duration of ART exposure prior to resistance testing there was a significant increase in treatment exposure for NNRTIs, PIs and INIs whereas NRTI exposure and the complete duration of treatment exposure increased without a level of significance. Therefore the highly significant decline in ADR observed among treated patients with available resistance test was not influenced by a declining duration of previous ART exposure or by increasing ART interruptions over time in the study population. In this study population PI exposure increased significantly over time. In contrast, PI associated HIV drug resistance in treated patients declined significantly. The enhanced use of PI containing regimens and the increasing variety of second generation PIs with higher resistance barriers might influence this phenomenon [26]. Although the proportion of ADR was high in treated patients, the majority of patients showed viral load measurements under the detection limit subsequent to the last resistance test. In view of the preventive effect of ART, these subjects are unlikely to contribute substantially to onward transmission of resistant HIV strains. The proportion of patients who were successfully treated did not differ between those with resistant HIV and those with susceptible viral strains, reflecting a sufficient number of antiretroviral treatment options and a high expertise in tailored individual treatment of HIV and careful monitoring of antiretroviral treatment for the majority of patients with ADR in this study. Since the introduction of the remuneration of HIV resistance testing by statutory health insurances in Germany in 2005, more and more patients were tested in clinical routine assuming that this have influenced the decline of ADR in the treated study population [7]. A significant increase in the proportion of patients who were resistance tested before ART initiation as well as in case of treatment failure was observed in this study population. However, the figures are still lower than expected if all patients are tested before ART start or in case of treatment failure as recommended in the guidelines. The proportion of patients tested before ART over calendar year as well as the proportion of patients failing therapy undergoing resistance testing may influence the proportion of observed HIV drug resistance in a study population. Perhaps the selection among persons before ART start, where we observe a rather consistent trend of TDR, was not as large as among treated patients. It is conceivable that there was a stronger selection bias to test patients with more problematic courses of therapy and thus towards cases with higher probability of HIV drug resistance in the initial years before statutory introduction of resistance testing in 2005.

Currently only HIV resistance data from long term observational studies in different study populations before ART initiation is available in Germany [8], [9], [19]. Therefore the ClinSurv- HIV Drug Resistance Study attempted to link HIV viral sequence data with epidemiological and treatment data to describe daily clinical resistance testing practices and the proportion of HIV drug resistance for both ART naïve patients as well as for treatment experienced patients in a subset of a large cohort study of people infected with HIV in Germany.

## Limitations

This study has some limitations. First, it is a convenience sample of data collected from 5 urban sites of Germany. Therefore, the study is only able to describe the situation based on the information that has been provided by those study centers, and did not reflect the overall situation for Germany. Furthermore, the data was collected over time periods where resistance testing became more widely used in routine clinical care. As we see the majority of the tests were performed after statutory introduction of resistance testing in 2005. Since the proportion of patients tested is correlated with the proportion of observed cases this is important to keep in mind in view of the findings. In addition, certain information is not retrieved, such as the reasons why, despite indication, no test was performed or the reasons for resistance testing, e.g. in view of future treatment options after interruption or due to an actual treatment failure. Evidence exists that in the participating centers more HIV drug resistance tests were performed than we observed by recording the data. It might be that resistance tests were carried out in other laboratories that we have not reached.

However, this report is the first description of HIV drug resistance for both ART naïve and treatment experienced persons infected with HIV in Germany. With all constraints, it is nevertheless one of the most comprehensive reports currently available and gives insight into clinical resistance testing practices and prevalence of HIV drug resistance of people infected with HIV in Germany.
